# G2c-Lineage Dominance and S1 Epitope-Glycan Drift of Porcine Epidemic Diarrhea Virus in Guangdong Province, China, 2022–2024

**DOI:** 10.3390/vetsci12111056

**Published:** 2025-11-02

**Authors:** Liangzong Huang, Liming Yan, Mengyi Zeng, Jianhui Yao, Jiaqi Hu, Weilin Zhong, Lewen Su, Guangzhi Yan, Shengnan Chen, Yuhan Huang, Mingjie Liu

**Affiliations:** 1 College of Animal Science and Technology, Foshan University, Foshan 528000, China; 13580781773@163.com (L.Y.);; 2 Guangdong Findergene Biotechnology Co., Ltd., Foshan 528000, China

**Keywords:** porcine epidemic diarrhea virus, S1 glycoprotein, neutralizing epitopes, glycosylation shift, G2c lineage

## Abstract

Porcine epidemic diarrhea (PED), a severe and highly contagious disease induced by porcine epidemic diarrhea virus (PEDV), impacts pigs across all age groups but has a particularly high lethality in neonatal piglets, with mortality rates reaching 80 to 100%, leading to substantial economic losses in the swine industry. In this investigation, 128 intestinal samples obtained from 65 large-scale pig farms in eight prefectures of Guangdong Province were screened by RT-qPCR between 2022 and 2024. Of these, 50 samples (39.06%) tested positive for Porcine Epidemic Diarrhea Virus (PEDV). The complete S1 genes of 31 representative strains were sequenced. Phylogenetic analysis revealed G2c as the exclusive dominant lineage (29/31, 93.6%), with single representatives of G2a and G2d. Nucleotide identity among the local strains ranged from 88.9 to 100% and 88.1 to 93.5% to prototype CV777 and from 91.2 to 99.1% to vaccine strain AJ1102. The COE neutralizing epitope (aa 499–638) carried 26 substitutions versus AJ1102; T499I/S, A520S/L, F539L, K566N and F615L were most prevalent. The SS2 epitope was fully conserved, whereas SS6 showed three low-frequency changes (S766P, S769F, G770V). Six distinct N-glycosylation patterns were identified relative to AJ1102. The predominance of G2c, accompanied by marked epitope drift and altered glycosylation, indicates the need for further investigation into vaccine efficacy. Continuous surveillance and the careful evaluation of G2c-based vaccine candidates are warranted.

## Simple Summary

This study investigated the genetic characteristics and antigenic drift of porcine epidemic diarrhea virus (PEDV) strains circulating in Guangdong Province, China, between 2022 and 2024. Among 128 samples collected from diarrheic piglets, 50 tested positive for PEDV. Phylogenetic analysis revealed that G2c is the dominant lineage (93.6%), with G2a and G2d present at low frequencies. Key neutralizing epitopes, particularly the COE epitope, showed significant amino acid substitutions compared to the vaccine strain AJ1102. Additionally, dynamic changes in N-glycosylation patterns were observed, including the frequent loss of N510 and sporadic acquisition of N340 or N127. These findings highlight the need for further evaluation of vaccine efficacy and continuous genomic surveillance to address emerging PEDV variants.

## 1. Introduction

Porcine epidemic diarrhea (PED) is an acute enteric infectious disease caused by porcine epidemic diarrhea virus (PEDV), characterized by high mortality rates (up to 80–100%) in suckling piglets, posing a persistent threat to the global swine industry [1,2,3]. First reported in the United Kingdom in 1971 [4], PEDV reached China in 1984, where the classical G1a strain CV777 was isolated and successfully controlled by homologous vaccines [5]. In the winter of 2010, the nationwide emergence of highly virulent G2-genotype porcine epidemic diarrhea virus (PEDV) strains constituted a critical epidemiological inflection point, eliciting 100% mortality in suckling piglets ≤ 7 days of age and irrevocably transforming the PED enzootic pattern in China [6,7].

PEDV, a member of the genus Alphacoronavirus, contains a 28 kb RNA genome of positive sense. This genome encodes the typical structural proteins, including spike (S), envelope (E), membrane (M), and nucleocapsid (N), along with the accessory ORF3 [8,9]. The S glycoprotein is the principal neutralizing antigen and is cleaved into S1 (aa 1–726, receptor-binding) and S2 (aa 727–1383, membrane-fusion) subunits [10,11]. Within S1, three linear neutralizing epitopes have been mapped: COE (aa 499–638), SS2 (aa 748–755), and SS6 (aa 764–771). Among these neutralizing epitopes, the COE epitope is responsible for recognizing and binding cellular targets and is an important target of the host antibody response and regarded as the primary target for subunit vaccine development against PEDV infection [12]. SS2 and SS6 are two B cell antigenic epitopes of PEDV S protein [13]. Mutations within these epitopes directly modulate viral antigenicity and vaccine efficacy [14,15]. N-glycosylation is an essential co-and posttranslational protein modification. It has a significant impact on both physicochemical properties and biological functions. It plays a significant role in protein folding and quality control, glycoprotein interaction, signal transduction, viral attachment, and immune response to infection [16].

Phylogenetically, PEDV is divided into two major genogroups: G1 (classical) and G2 (variant). The G2 genogroup, dominant since 2010, is further split into G2a, G2b, G2c, and G2d sublineages [17]. Because inter-genogroup divergence is concentrated in the S1 subunit of the spike glycoprotein, this region serves as the primary molecular marker for surveillance and for assessing vaccine-match efficacy.

Between 2022 and 2024, intestinal and fecal specimens were systematically collected from diarrheic piglets on 65 large-scale commercial farms located in eight prefectures of Guangdong Province, China. The primary objective of this study was to investigate the genetic characteristics and antigenic compatibility of the predominant porcine epidemic diarrhea virus (PEDV) strains circulating in the region with current vaccine candidates. By focusing on the S1 glycoprotein, a critical component for neutralizing antibodies and a key molecular marker for vaccine efficacy, we sought to provide insights into the prevailing genotype and potential antigenic drift. This information is vital for refining regional vaccination strategies and identifying suitable vaccine candidates for future development.

## 2. Materials and Methods

### 2.1. Sample Collection and Processing

From 2022 to 2024, a total of 128 clinical samples, including intestinal tissues, fecal matter, and rectal swabs, were obtained from piglets displaying diarrhea. These samples were sourced from 65 large-scale pig farms located across eight prefectures (Foshan, Qingyuan, Heyuan, Jiangmen, Zhanjiang, Maoming, Meizhou, and Shaoguan) in Guangdong Province, China. Each specimen was suspended in sterile phosphate-buffered saline (PBS) (Sevier, Wuhan, China), homogenized (LIUYI grinder, Beijing, China) and clarified by centrifugation at 8000 rpm for 5 min, except for the rectal swab samples, which were directly processed in PBS without homogenization and centrifugation. The resulting supernatants were stored at –80 °C until nucleic-acid extraction.

### 2.2. Primer and Probe Design

The TaqMan RT-qPCR primers and probe sequences for PEDV were designed according to the standard SN/T1699-2017 [18]. For amplification of the full-length S1 gene (2.2 kb), a primer pair was designed in SnapGene (Version 6.0.2) after alignment of all available PEDV G1 and G2 sequences in GenBank: 5′-CCGGAATTCATGAAGTCTTTAACCTACTTCTGG-3′ (forward, EcoRI site underlined) and 5′-GGGAAGCTTAATACTCATACTAAAGTTGGTGGGA-3′ (reverse, HindIII site underlined). All oligonucleotides were synthesized by Sangon Biotech (Shanghai, China) and diluted to 10 µM working solutions.

### 2.3. Viral RNA Isolation and RT-qPCR Detection

Viral RNA was extracted from 200 μL of each clarified homogenate using the Vazyme FastPure Viral DNA/RNA Mini Kit (RC311) on the Vazyme automated workstation (Nanjing, China), and PEDV RNA was detected by one-step RT-qPCR with the Vazyme HiScript III U+ Probe Kit (Q225) on a Roche LightCycler 480 system (Basel, Switzerland) according to the manufacturers’ protocols.

### 2.4. RT-PCR and Sequencing

For each PEDV-positive sample, viral RNA was reverse-transcribed into cDNA with the Vazyme HiScript^®^ II 1st Strand cDNA Synthesis Kit (R211), and the entire S1 gene was amplified using 2× Phanta Max Master Mix (P515) and the gene-specific primers described in Section 2.2. The PCR reaction program included an initial denaturation step at 95 °C for 3 min, followed by 35 cycles of 95 °C for 15 s (denaturation), 58 °C for 15 s (annealing), and 72 °C for 2 min (extension), and a final extension at 72 °C for 5 min. Amplicons were examined on 2% agarose gels against DNA ladders; products of the expected size were excised, purified, and submitted to Sangon Biotech (Shanghai, China) for bidirectional Sanger sequencing.

### 2.5. Genetic Evolution and Amino Acid Sequence Variation Analysis of PEDV S1

The S1 gene sequences were assembled and corrected using SeqMan in the DNAstar software (version 7.1; DNAstar, Madison, WI, USA). These sequences were compared with 32 representative PEDV reference strains (covering G1–G2 subtypes) from GenBank. A phylogenetic tree was constructed using the neighbor-joining method in MEGA software (version 11.0) with 1000 bootstrap replicates and visualized using the Chiplot online platform to determine the genotype affiliation and evolutionary branches of the epidemic strains [19]. Nucleotide similarity and amino acid sequence were evaluated via the Clustal W approach within the MegAlign tool of DNAStar software. Amino acid sequence variations in the S1 protein were annotated, focusing on mutation sites within the COE, SS2, and SS6 epitope regions (COE, SS2, and SS6 epitopes in the S1 protein were mapped and analyzed based on previously established conserved neutralizing epitope regions). Additionally, online tools were employed to predict changes in N-glycosylation sites (https://services.healthtech.dtu.dk/services/NetNGlyc-1.0; accessed on 8 July 2025). N-glycosylation sites with a score of “++” or above were regarded as highly specific [20].

## 3. Results

### 3.1. RT-qPCR Screening and S1-Gene Acquisition

Of the 128 clinical specimens, 50 (39.06%) tested positive for PEDV by RT-qPCR. The S1 gene was successfully amplified from these positive samples, yielding 31 high-quality sequences after Sanger sequencing and subsequent assembly using DNAStar SeqMan Pro.

### 3.2. Genotyping and Genetic Distance of PEDV S1 Sequences

**Figure 1:**
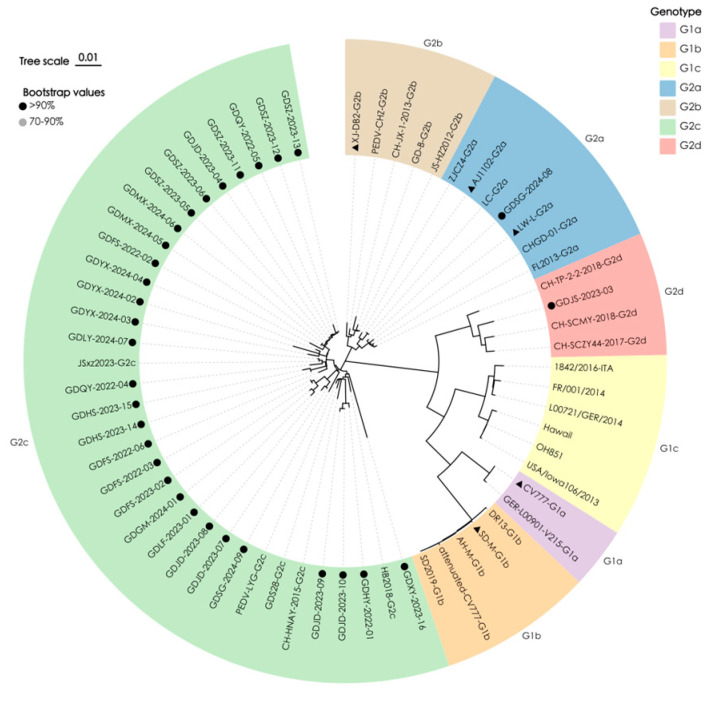
Neighbor-joining phylogeny of PEDV S1 genes. The tree incorporates 31 Guangdong isolates (●) and 32 global reference strains; vaccine strains are indicated (▲). Branches are color-coded by genotype (G1a–c, G2a–d). Analysis was performed in MEGA 11 with 1000 bootstrap replicates; bootstrap values ≥ 70% are shown at the nodes.

Phylogenetic analysis classified the 31 Guangdong S1 sequences into three genotypes: 29 strains (93.6%) clustered with G2c references (GDS28, CH-HNAY-2015, HB2018, PEDV-LYG); one strain (GDJS-2023-03) grouped with G2d references (CH-TP-2-2-2018, CH-SCZY44-2017, CH-SCMY-2018); and one strain (GDSG-2024-08) aligned with G2a references (LW-L, CHGD-01, LC) (Figure 1).

Pairwise identities among the 31 local sequences ranged 88.9–100%. These strains showed 92.1% to 93.2% similarity to the classical strain CV777. Compared with vaccine strains AJ1102, XJ-DB2-G2b, LW-L-G2a, and SD-M-G1b, the similarities were 91.2–99.1%, 91.3–98.2%, 91.2–99.3%, and 86.8–93.0%, respectively. Within the dominant G2c clade, local strains shared 94.6–100% identity with each other and 96.0–99.9% with G2c references. The sole G2d isolate displayed 98.6–98.9% identity to G2d references, whereas the G2a isolate showed 99.0–99.8% identity to G2a references. Collectively, G2c has become the prevailing genotype in Guangdong, while G2a and G2d persist at low frequencies.

### 3.3. Variation Analysis of Key Neutralizing Epitopes in PEDV S1 Protein

Compared to the vaccine strain AJ1102, the 29 G2c isolates exhibited 26 amino-acid substitutions within the COE epitope, with recurrent changes at T499I/S, A520S/L, F539L, K566N and F615L. Strain GDXY-2023-16 displayed a tandem substitution (AF520-521LL), while GDFS-2022-02 carried SK586-587GR. The SS2 epitope was completely conserved across all local strains, whereas SS6 showed only sporadic mutations (S767P, S769F or G770V) in one to three isolates. The sole G2d isolate differed by only the A520S substitution, whereas the single G2a isolate exhibited complete identity to strain AJ1102 across all three neutralizing epitopes examined. Analysis of N-glycosylation sites revealed that AJ1102 harbors motifs at positions 62, 118, 212, 320, 347 and 510. The G2d isolate GDJS-2023-03 lacked sites at 62, 118 and 510 but acquired novel motifs at 127 and 347. The G2a isolate GDSD-2024-08 shared all sites with AJ1102 except for the loss of 347. Among the G2c strains, 15 isolates lacked only residue 510, ten lost both 347 and 510, GDLF-2023-01 and GDGM-2024-01 further lost site 62, and GDHS-2023-14/-15 uniquely gained site 340 while losing 510 (Table 1).

## 4. Discussion

Since 2010, highly virulent G2-subtype PEDV variants have disseminated worldwide, inflicting severe losses on swine production. The virus’s high evolutionary rate continuously erodes vaccine efficacy, making sustained surveillance indispensable. In this study, we collected 128 samples from 65 farms experiencing diarrhea outbreaks. This sampling was part of outbreak investigations rather than systematic surveillance. While this approach enabled us to focus on the genetic characteristics of outbreak cases and revealed key features of viral dynamics during outbreaks, we acknowledge that it may introduce sampling bias. This bias could limit the generalizability of the reported 39.06% positivity rate. Nevertheless, the findings remain relevant for informing regional disease surveillance and control strategies. Our phylogenetic analysis resolved Guangdong sequences into three G2 sublineages, with G2c accounting for 93.6% (29/31) of isolates; this prevalence is consistent with previously reported national trends [21,22,23,24]. The sporadic detection of G2d, previously reported only in Sichuan, Hebei and Henan [17], implies interstate spread via live-animal transport and underscores the need for real-time genomic tracking.

Although the field isolates share 91.2–99.1% nucleotide identity with the G2a-based vaccine AJ1102—substantially higher than with classical CV777—marked antigenic drift has accumulated within the S1 gene. Twenty-six amino-acid substitutions, concentrated in the COE neutralizing epitope, include recurrent changes (A520S/L, F539L, K566N, F615L) adjacent to receptor-binding motifs that are predicted to modulate neutralization sensitivity [25]. Two isolates harbor tandem mutations (AF520-521LL; SK586-587GR) that may impact the three-dimensional structure of the S1 protein. These structural changes could lead to the alterations in antigenic epitopes [26], which may not be recognized by vaccines designed based on the original AJ1102 strain. This highlights the urgency for functional validation of these genetic changes to assess their actual impact on vaccine protection.

In addition to amino-acid substitutions, S-protein glycosylation profoundly influences coronavirus infectivity and immunogenicity [27,28,29,30]. Our analysis of N-glycosylation site variations reveals a dynamic pattern of glycan site gain and loss among the prevailing G2c strains. By comparing the frequency of N-glycosylation site acquisition and loss in PEDV strains at different time points, we infer the existence of glycan drift. Specifically, we observed universal loss of N510, frequent loss of N347, and sporadic acquisition of N340 or N127. These dynamic changes may reflect the virus’s evolutionary strategy to modulate its glycan shield under immune pressure, thereby influencing its antigenicity and neutralizing properties.

For example, seven N-glycosylation sites in the SARS-CoV-2 S protein have mutated, significantly impacting its interaction with ACE2 and its transmission ability. Mutations at sites N227 and N699 enhance viral transmissibility, while deletions at N331 and N343 reduce it [31,32]. Deletion of N234 increases the virus’s antagonism to neutralizing antibodies, and deletion of N165 makes the virus more sensitive to them [33]. Similarly, in PEDV, research shows that removing the N-glycosylation sites at positions 118, 216, and 726 can reduce viral replication and plaque size, and eliminating the sites at positions 514 and 556 in the PEDV S protein (N514G and N556G) can enhance IgG and neutralizing antibody titers without changing PEDV pathogenicity [34,35]. These examples show that changes in N-glycosylation sites in local PEDV strains may enhance the virus’s immune evasion capability, increasing the risk of vaccine immune failure and complicating PED prevention and control. Future research should further explore the functional implications of these glycan changes on vaccine efficacy.

While the S1 gene is a critical region for understanding antigenic variation and viral evolution, whole-genome sequencing would provide a more complete picture of viral evolution. This includes the potential identification of recombination events, which are common in coronaviruses and can significantly impact viral fitness and antigenicity. Further research should consider whole-genome sequencing to fully understand the genetic diversity and evolutionary dynamics of PEDV. Additionally, functional immunological assays like virus neutralization assays, which test field isolates against vaccine-induced sera, can evaluate the influences of genetic drift on vaccine efficacy.

## 5. Conclusions

Guangdong’s PEDV population is now dominated by G2c strains that carry key substitutions in the COE epitope and a recurrent N510 glycan deletion—features that may influence vaccine efficacy and warrant further functional studies. Comprehensive evaluation of G2c-matched vaccine seeds and continual genomic surveillance are essential for controlling PEDV in the region.

## Figures and Tables

**Table 1:** Variation in highly specific N-glycosylation sites in the S protein of different Subgroup strains in comparison to CV777 and AJ1102 vaccine strain.

Strains	High-Specificity N-Glycosylation Sites								
	62	118	127	212	320	340	347	510	552
CV777-G1a -AF353511	-	-	NKTL	NVTS	NDTS	-	-	NITV	NVTN
GDJS-2023-03 -G2d	-	-	NKTL	NVTS	NDTS	-	NSSN	-	-
AJ1102-G2a -JX188454	NSTW	NATA	-	NVTS	NDTS	-	NSSD	NITV	-
GDSD-2024-08- G2a	NSTW	NATA	-	NVTS	NDTS	-	-	NITV	-
GDLF-2023-01	-	NATA	-	NVTS	NDTS	-	NSSN	-	-
GDGM-2024-01	-	NATA	-	NVTS	NDTS	-	NSSN	-	-
GDHS-2023-14	NSTW	NATA	-	NVTS	NDTS	NLSF	-	-	-
GDHS-2023-15	NSTW	NATA	-	NVTS	NDTS	NLSF	-	-	-
GDQY-2022-04	NSTW	NATA	-	NVTS	NDTS	-	-	-	-
GDFS-2022-06	NSTW	NATA	-	NVTS	NDTS	-	-	-	-
GDJD-2023-04	NSTW	NATA	-	NVTS	NDTS	-	-	-	-
GDSZ-2023-05	NSTW	NATA	-	NVTS	NDTS	-	-	-	-
GDSZ-2023-06	NSTW	NATA	-	NVTS	NDTS	-	-	-	-
GDSZ-2023-12	NSTW	NATA	-	NVTS	NDTS	-	-	-	-
GDYX-2024-02	NSTW	NATA	-	NVTS	NDTS	-	-	-	-
GDYX-2024-03	NSTW	NATA	-	NVTS	NDTS	-	-	-	-
GDYX-2024-04	NSTW	NATA	-	NVTS	NDTS	-	-	-	-
GDLY-2024-07	NSTW	NATA	-	NVTS	NDTS	-	-	-	-
GDHY-2022-01	NSTW	NATA	-	NVTS	NDTS	-	NSSN	-	-
GDFS-2022-02	NSTW	NATA	-	NVTS	NDTS	-	NSSN	-	-
GDFS-2022-03	NSTW	NATA	-	NVTS	NDTS	-	NSSN	-	-
GDQY-2022-05	NSTW	NATA	-	NVTS	NDTS	-	NSSN	-	-
GDFS-2023-02	NSTW	NATA	-	NVTS	NDTS	-	NSSN	-	-
GDJD-2023-07	NSTW	NATA	-	NVTS	NDTS	-	NSSN	-	-
GDJD-2023-08	NSTW	NATA	-	NVTS	NDTS	-	NSSN	-	-
GDJD-2023-09	NSTW	NATA	-	NVTS	NDTS	-	NSSN	-	-
GDJD-2023-10	NSTW	NATA	-	NVTS	NDTS	-	NSSN	-	-
GDSZ-2023-11	NSTW	NATA	-	NVTS	NDTS	-	NSSN	-	-
GDSZ-2023-13	NSTW	NATA	-	NVTS	NDTS	-	NSSN	-	-
GDXY-2023-16	NSTW	NATA	-	NVTS	NDTS	-	NSSN	-	-
GDMX-2024-05	NSTW	NATA	-	NVTS	NDTS	-	NSSD	-	-
GDMX-2024-06	NSTW	NATA	-	NVTS	NDTS	-	NSSD	-	-
GDSG-2024-09	NSTW	NATA	-	NVTS	NDTS	-	NSSN	-	-

## Data Availability

The original contributions presented in this study are included in the article material. Further inquiries can be directed to the corresponding author.
